# Nuclear TRADD prevents DNA damage-mediated death by facilitating non-homologous end-joining repair

**DOI:** 10.1038/s41598-017-03211-z

**Published:** 2017-06-13

**Authors:** Gi-Bang Koo, Jae-Hoon Ji, Hyeseong Cho, Michael J. Morgan, You-Sun Kim

**Affiliations:** 1 Department of Biochemistry, Ajou University School of Medicine, Suwon, Gyeonggi 16499 Republic of Korea; 2 Department of Biomedical Sciences, graduate School, Ajou University, Suwon, Gyeonggi 16499 Republic of Korea; 3 Genomic Instability Research Center, Ajou University School of Medicine, Suwon, Gyeonggi 16499 Republic of Korea; 4 Department of Pharmacology, University of Colorado School of Medicine, Aurora, Colorado 80045 USA

## Abstract

TNF receptor-associated death domain (TRADD) is an essential mediator of TNF receptor signaling, and serves as an adaptor to recruit other effectors. TRADD has been shown to cycle between the cytoplasm and nucleus due to its nuclear localization (NLS) and export sequences (NES). However, the underlying function of nuclear TRADD is poorly understood. Here we demonstrate that cytoplasmic TRADD translocates to DNA double-strand break sites (DSBs) during the DNA damage response (DDR). Deficiency of TRADD or its sequestration in cytosol leads to accumulation of γH2AX-positive foci in response to DNA damage, which is reversed by nuclear TRADD expression. TRADD facilitates non-homologous end-joining (NHEJ) by recruiting NHEJ repair factors 53BP1 and Ku70/80 complex, whereas TRADD is dispensable for homologous recombination (HR) repair. Finally, an impaired nuclear localization of TRADD triggers cell death through the persistent activation of JNK and accumulation of reactive oxygen species (ROS). Thus, our findings suggest that translocation of TRADD to DSBs into the nucleus contributes to cell survival in response to DNA damage through an activation of DNA damage repair.

## Introduction

TNF Receptor 1 (TNFR1) signaling has been studied intensively using the knockout mouse models including knockout of RIP1 (Receptor-interacting protein 1), TRAF2 (TNFR-associated factor 2), FADD (FAS-associated death domain protein) and TRADD (TNFR1-associated death domain protein). TRADD is required for TNFR1-mediated ‘downstream’ signaling events such as activation of the NF-κB and MAPK as well as cell death^1, 2^. Generation of TRADD-deficient mice showed that TRADD has critical functions in TNFR1, TLR (Toll-like receptor) and TRAIL (TNF-related apoptosis-inducing ligand) signaling by orchestrating the formation of signaling complexes^2, 3^. In death receptor-mediated signaling pathways, TRADD serves as adaptor molecule to recruit other effectors^4^, but also has functions in mediating other various biological processes. For instance, TRADD is also crucial for the Retinoic acid Inducible Gene-1 (RIG-1) helicase antiviral pathway through its recruitment to Cardif to regulate inflammatory responses^5^.

The human TRADD gene at chromosome 16q22.1 shows frequent loss-of-heterozygosity (LOH) in various tumor types, indicating that loss of TRADD may promote tumorigenesis^6, 7^. Consistent with this, TRADD-deficient mice exhibit enhanced tumor formation in DMBA/TPA-induced skin carcinogenesis^8^. Although TRADD has largely been studied as a cytoplasmic adaptor in death receptor signaling, TRADD is known to have a nuclear export signal (NES) at amino acid 147–163 and a nuclear localization signal (NLS) at amino acid 229–242, which allows shuttling between the nucleus and the cytoplasm^9^. It has been recently reported that nuclear localization of TRADD promoted p19^Arf^ protein stability and tumor suppression by regulating ULF-dependent p19^Arf^ ubiquitylation in a mouse model of skin cancer^8^. However, TRADD is expressed at high levels in GBM (Glioblastoma multiforme) where it is detected in both the cytoplasm and the nucleus^10^, and silencing of TRADD in glioma cells resulted in increased sensitivity to TMZ (Temozolomide) by regulating NF-κB, suggesting that cytoplasmic TRADD is a key driver of NF-κB activation in GBM. Therefore, TRADD may have dual pro-cancer and anti-cancer functions, depending on cellular localization.

DNA double-strand breaks (DSBs) are the most deleterious of DNA lesions, and, if left unrepaired, may have severe consequences for cell survival, as they lead to chromosome aberrations, genomic instability, or cell death. Various physical, chemical, and biological factors are involved in generation of DSB^11^. DNA can be damaged by exogenous agents such as radiation, X-ray, UV, alkylating agents, as well as by the by-products from endogenous processes such as reactive oxygen and nitrogen species. DNA repair proteins often localize in the nucleus after DNA damage in order to modulate DNA damage responses (DDRs); these proteins often contain a NLS and NES sequences that cause the protein to shuttle in and out of the nucleus^12, 13^. Therefore, we investigated whether TRADD translocation from the cytoplasm into the nucleus is associated with a DNA damage response. We found that, upon DNA damage, TRADD moves to the nucleus and modulates the non-homologous end-joining (NHEJ) DNA repair pathway. Deficiency of TRADD during the DNA damage response causes increased reactive oxygen species (ROS) and persistent activation of the stress-activated kinase, JNK, leading to cell death. Our data suggest that TRADD is a potential target for initiating cancer cell death in response to therapeutic DNA-damaging agents.

## Results

### TRADD is involved in the hydrogen peroxide-induced DNA damage response

**Figure 1:**
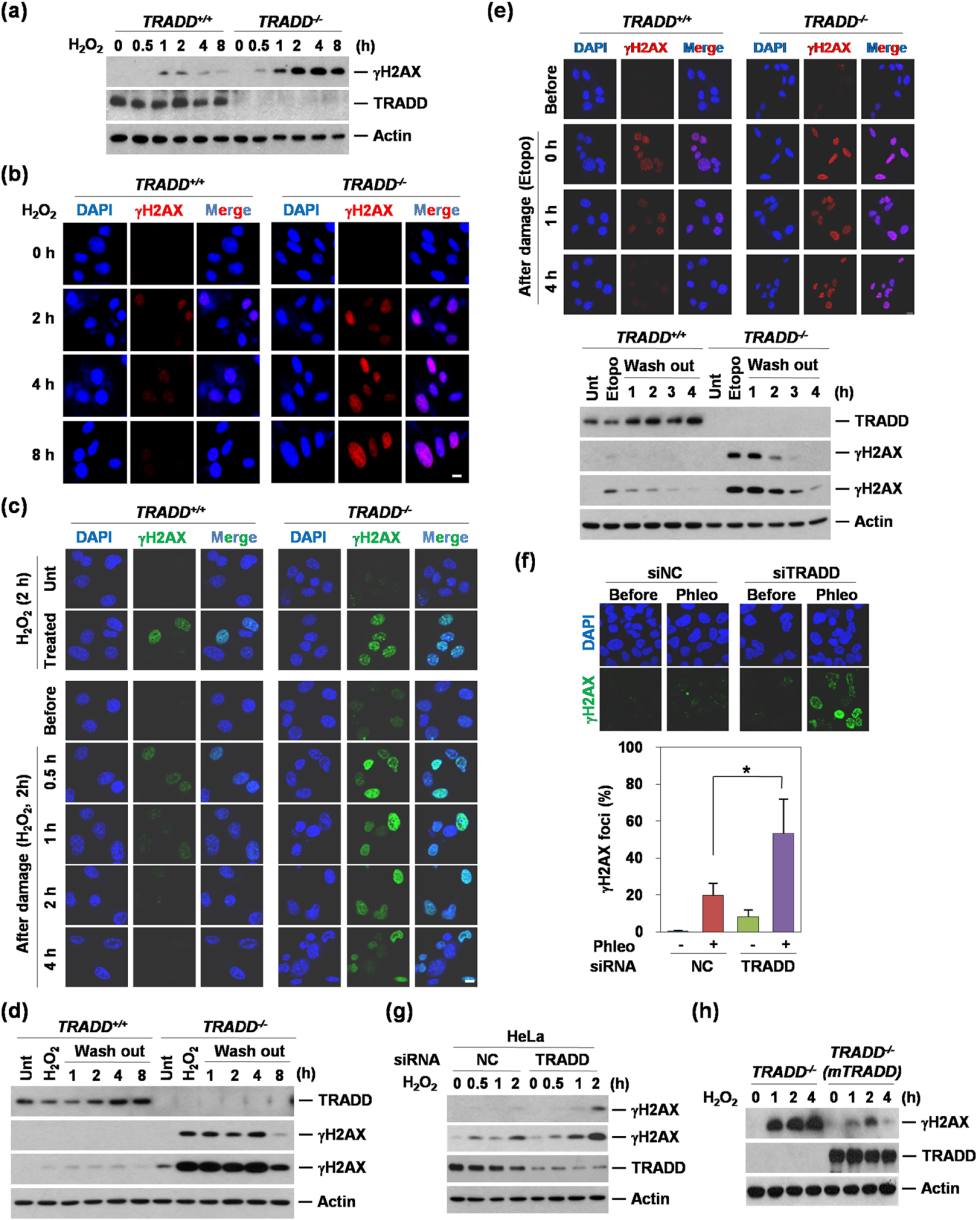
Deficiency of TRADD induces impaired DNA damage response. (**a**) Western blotting analysis shows blotting for γH2AX, TRADD, and Actin in TRADD^+/+^ and TRADD^−/−^ MEF cells treated with H_2_O_2_ in time-dependent manner (0.5 mM). (**b**) γH2AX foci (Red) were analyzed in TRADD^+/+^ and TRADD^−/−^ MEF cells treated with H_2_O_2_ (0.5 mM) by Immunofluorescense as described in A. Scale bars, 10 μm. (**c**) Immunofluorescence analyses of γH2AX (Green) in H_2_O_2_ (0.5 mM) treated TRADD^+/+^ and TRADD^−/−^MEF for 2 hours (upper panels) and release from H_2_O_2_ treated TRADD^+/+^ and TRADD^−/−^ MEF for 4 hours (lower panels). Cells were stained with anti-γH2AX (Green) and DAPI (Blue). Scale bars, 10 μm. (**d**) Western blotting analysis shows results consistent with immunofluorescence as described in (**c**). (**e**) After cells were treated with etoposide (25 μM) for 1 hour, TRADD^+/+^ and TRADD^−/−^MEF replaced with fresh media. Cells were stained with anti-γH2AX (Red) and DAPI (Blue). Western blotting analysis (lower panel) shows the consistent results with immunofluorescence. Scale bars, 10 μm. (**f**) Quantitative analysis of γH2AX foci was conducted in TRADD knock-downed U2OS cells. After TRADD knockdown, cells were treated with phleomycin (Phleo) and then stained with γH2AX antibody. *P < 0.05 (Student’ s t-test). (**g**) Transient knockdown of TRADD induces unrepaired DNA damage in HeLa cells. Western blot analysis shows γH2AX status in response to H_2_O_2_ in TRADD KD HeLa cells. Cells were transfected with siRNA TRADD or siRNA negative control (NC), respectively. After 48 hours, the cells were continuously treated with H_2_O_2_ (0.5 mM). The whole cell lysates were analysed by western blot as using indicated antibodies. (**h**) Reconstitution of TRADD in TRADD^−/−^ MEFs. Western blotting analysis shows γH2AX expression in response to continuous treatment with H_2_O_2_ (0.5 mM) in different time points.

Although the cytoplasmic functions of TRADD have been investigated intensively, much less is known about its function in the nucleus. To investigate this role, we first tested whether TRADD status affects the cellular response to DNA damage induced by hydrogen peroxide (H_2_O_2_), which generates hydroxyl radicals in the presence of transition metal ions, and can diffuse into the nucleus to cause DNA strand breaks. We treated TRADD wild type (TRADD^+/+^) and TRADD knockout (TRADD^−/−^) MEFs with H_2_O_2_ and followed the phosphorylation (at Ser 139) of histone H2AX (γH2AX), which is one of the major markers for DNA double-strand breaks (DSBs)^14^. Deficiency of TRADD potentiated the appearance of γH2AX in response to H_2_O_2_ in both western blotting and immunofluorescence staining (Fig. 1a and b), indicating that TRADD may be involved in the DNA damage response (DDR). In further experiments, cell growth media were replaced after 2 hours of H_2_O_2_ treatment of the TRADD^+/+^ and TRADD^−/−^ MEFs, to prevent further DNA damage. In the presence of TRADD, γH2AX was significantly reduced or gone 4 hours later; however, γH2AX detection largely persisted in TRADD^−/−^ MEFs (Fig. 1c and d). Hydrogen peroxide induces a large variety of different cellular damage to DNA and proteins. To verify the involvement of TRADD in DNA damage responses, we treated cells with specific DNA damage-inducing agents. Deficiency of TRADD potentiated the appearance of γH2AX in response to the alkylating agent N-methyl-N′-nitro-N′-nitrosoguanidine (MNNG); doxorubicin also had a similar pronounced effect on γH2AX in TRADD-deficient cells (Figure S1a). As expected, various DNA damage-inducing agents including doxorubicin (Doxo), etoposide (Etopo), camptothecin (Cpt), and hydroxyurea (Hu) resulted in a differential increase of γH2AX in TRADD^−/−^ MEFs, but cisplatin (CDDP) had a minimal effect on γH2AX (Figure S1b and c). Etoposide led to a particularly potent activation of γH2AX in TRADD^−/−^ MEFs compare to TRADD^+/+^ MEFs. To further investigate this effect, TRADD^+/+^ and TRADD^−/−^ MEFs were treated with etoposide and 1 hour later, the media were replaced with fresh media to prevent further DNA damage. In the presence of TRADD, γH2AX foci were significantly reduced compared with TRADD^−/−^ MEFs, as were γH2AX detection by immunostaining and western blotting (Fig. 1e). To determine whether TRADD-deficiency sensitizes to DSBs in other cell types, we reduced TRADD expression in U2OS and HeLa cells by siRNA knock-down (KD). Increased γH2AX was further observed in TRADD KD U2OS cells treated with phleomycin (Phleo), a DSB-mimetic drug (Fig. 1f). In TRADD KD HeLa cells, γH2AX was substantially increased in response to H_2_O_2_ (Fig. 1g). Ectopic expression of TRADD (mTRADD) in TRADD^−/−^ MEFs inhibited the appearance of γH2AX in response to H_2_O_2_ (Fig. 1h), confirming that this response was specifically due to TRADD deficiency, and not an artifact of MEF cell line generation. To exclude the possibility that deletion of TRADD affected the cell cycle, we confirmed cell cycle alterations in TRADD knockdown U2OS cells, TRADD^+/+^ and TRADD^−/−^ MEFs. There were no cell cycle differences in TRADD deficient cells that might indirectly influence which DNA repair pathway could be utilized (Figure S1d and e). Taken together, these data suggest that TRADD is involved in the cellular response to DNA damage and that TRADD is either required for increased DNA repair and/or reduced DNA damage.

### Nuclear TRADD is necessary for response to DNA damage

**Figure 2:**
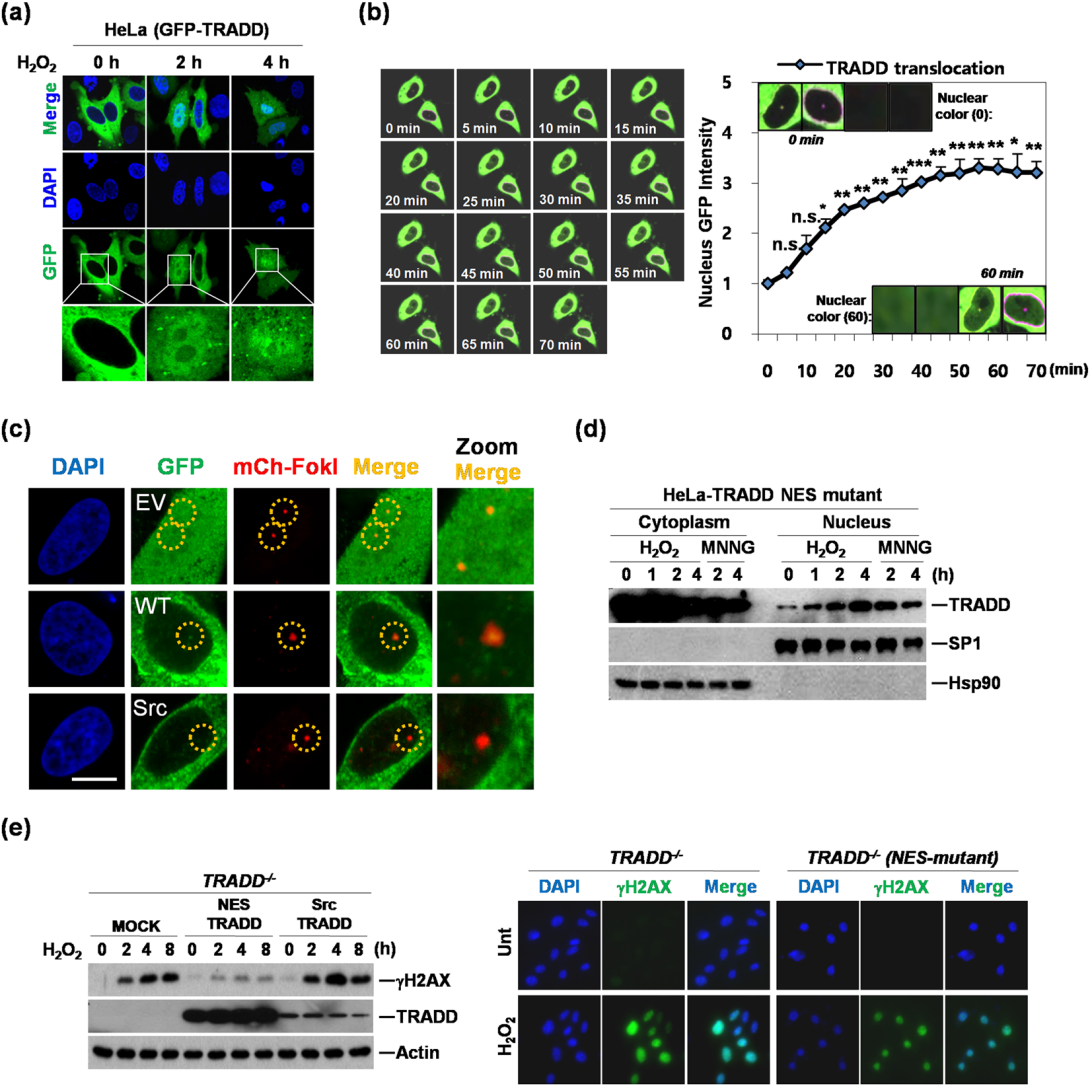
DNA damage induces nuclear translocation of TRADD. (**a**) HeLa cells were transiently transfected with GFP-TRADD and treated with H_2_O_2_ (0.5 mM) for indicated time points. Cells were analyzed by confocal fluorescence microscopy. (**b**) HeLa cells were transiently transfected with GFP-TRADD and treated with H_2_O_2_ (0.5 mM). After treatment, live cell Images were analyzed by confocal fluorescence microscopy for 70 minutes (left panel). Quantitative analysis of nuclear translocation of TRADD was measured by GFP intensity in the nucleus (right panel). *P < 0.05; **P < 0.01; ***P < 0.001; n.s., not significant (Student’s t-test). (**c**) Colocalization of GFP-TRADD and mCherry-FokI at single DNA double-strand break site. GFP empty vector (EV), GFP-TRADD wild type (WT), or GFP-TRADD Src mutant (SRC) was cotranfected with mCherry-FokI (mCh-FokI) nuclease into U2OS 2-6-3 cell lines. After 48 hr, cells were fixed and stained with DAPI for nuclear staining. Images were analyzed confocal microscope (Nikon A1). Scale bar, 10 μm. (**d**) HeLa cell were transiently transfected with NES mutant TRADD and treated with H_2_O_2_ (0.5 mM) or MNNG (0.25 mM) for indicated times. Cells were fractionated into cytoplasmic and nuclear fractions using an NE-PER fractionation kit. Anti-Hsp90 or anti-Sp1 used as a control for normalization of cytoplasm and nuclear lysates, respectively. (**e**) Western blotting analysis was conducted with lysates from TRADD^−/−^ (MOCK), NES-mutant TRADD (NES-TRADD) and Src-myristoylation-TRADD (Src-TRADD) in TRADD^−/−^ MEFs treated with H_2_O_2_ (0.5 mM) for indicated time periods (left panel). Expression of γH2AX was analyzed in TRADD^−/−^ and TRADD^−/−^ (NES-mutant TRADD) MEFs treated with H_2_O_2_ (0.5 mM) for 1 hour using immunofluorescence (right panels).

To monitor the translocalization of TRADD into the nucleus in response to DNA damage, we transfected a GFP-TRADD plasmid into HeLa cells. When cells were treated with hydrogen peroxide to induce DNA damage, GFP-TRADD translocated into the nucleus (Fig. 2a). This translocation began as early as 10–15 minutes post-treatment, and further increased in a time-dependent manner, as measured by time-lapse epifluorescent and confocal microscopy (Figs 2b and S2a). TRADD has an NLS and an NES, which causes shuttling between nucleus and cytosol^9^. To clarify the role of TRADD translocation into the nucleus, we expressed previously-generated TRADD constructs^9^ after testing their expression and localization. The NES mutant-TRADD localizes both in the nucleus and cytosol, but once imported into the nucleus, it cannot translocate to the cytosol, since it lacks an export sequence. Cytoplasmic-TRADD is a C-terminal deletion lacking the death domain (and the NLS) and localizes exclusively in the cytosol, while Src-myr-TRADD is largely confined to the plasma membrane by an ectopic SRC-myristoylation sequence. HeLa cells were transfected with different TRADD constructs and their localization was verified by fluorescence microscopy (Figure S2b). To determine whether TRADD was recruited specifically to DNA break sites, we used the mCherry-LacI-FokI endonuclease system, which targets mCherry-tagged FokI nuclease activity specifically to lac operator repeats^15^. In the presence of DNA damage, some GFP-fused exogenous wild type TRADD translocated into the nucleus and colocalized with the mCherry-FokI signal, whereas the Src-myr-TRADD mutant and GFP vector alone did not show any colocalization with mCherry-FokI (Fig. 2c), suggesting that cytosolic TRADD is recruited to the nucleus at DNA breakage sites upon DNA damage. Next, we tested again whether nuclear TRADD is essential for down-regulation of persistent γH2AX. We found that NES mutant-TRADD and endogenous TRADD accumulated in the nucleus after H_2_O_2_ or MNNG treatment, as measured by western blotting of nuclear and cytoplasmic fractions (Figs 2d and S2c). Furthermore, when TRADD^−/−^ MEFs were transfected with different TRADD plasmids (Figure S2d), reconstitution of NES mutant TRADD in TRADD^−/−^ MEFs repressed the increase of γH2AX and γH2AX foci in response to H_2_O_2_ treatment (Fig. 2e). However, Src-myr-TRADD, which is confined to the plasma membrane, had no effect on γH2AX status upon H_2_O_2_ treatment, indicating that that nuclear localization of TRADD is required for the increased DNA repair or reduced DNA damage.

### TRADD is required for the non-homologous end-joining pathway

**Figure 3:**
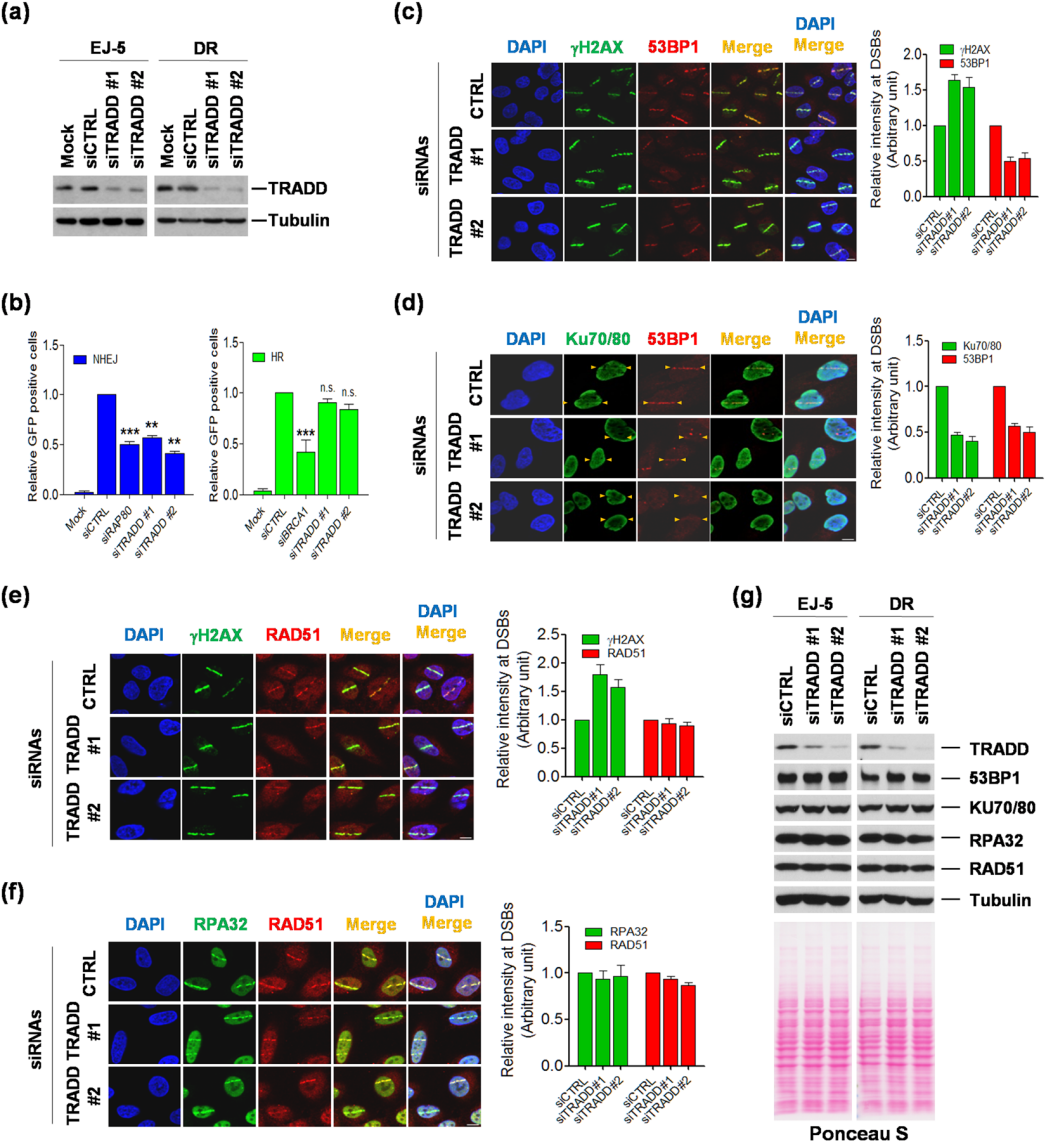
TRADD is required for non-homologous end-joining repair. (**a**) Knockdown efficacy for TRADD in DNA repair reporter cell lines EJ5 and DR. (**b**) After 48 hours transfection with TRADD, RPA80, or BRCA1 targeting siRNAs into reporter cell lines, each siRNA was again cotransfected with an I-*SceI* endonuclease construct. After 72 hours, GFP positive cells were analyzed with a flow cytometer (FACScan). **P < 0.01; ***P < 0.001; n.s., not significant (ANOVA). (**c**,**d**) After 1 hour with laser microirradiation, endogenous NHEJ repair factors were stained with each antibody at DNA break sites: 53BP1 (**c**); Ku70/80 and 53BP1 (**d**). Scale bars, 10 μm. (**e**,**f**) Endogenous HR repair factors were stained as described in **c**. RAD51 (**e**); RPA32 and RAD51 (**f**). γH2AX was used as a DNA damage marker at DNA break sites in (**c**) and (**e**). Scale bars, 10 μm. (**g**) The protein levels of repair factors in TRADD depletion. EJ-5 or DR cells were transfected with TRADD siRNAs (#1 or #2) or control siRNA. The levels of repair factors were detected by using target antibody, respectively. Total protein levels were verified with Ponceus S staining and tubulin antibody as a loading control.

There are two major DNA double-strand break repair pathways: non-homologous end-joining (NHEJ) and homologous recombination (HR) repair^16, 17^. Since deficiency of TRADD revealed the persistence of γH2AX propagation with DNA damaging agents, we sought to determine which of these pathways TRADD might participate in. Depletion of TRADD with two different siRNAs (TRADD#1 and TRADD#2) was done in EJ-5 and DR GFP DNA repair reporter cell lines (Fig. 3a). In EJ-5 cells, a promoter is separated from a GFP-coding sequence by a puro gene that has two I-*Sce*I sites that are in the same orientation at opposite ends, such that EJ repair restores GFP expression. In DR-GFP cells, the genome contains a full-length GFP mutated to contain an I-*Sce*I site, as well as a 5′ and a 3′-truncated GFP, which can then reconstitute GFP by HR machinery. After I-*Sce*I expression, it was apparent that TRADD was required for efficient NHEJ repair, whereas this phenomenon was dispensable for HR repair (Fig. 3b). TRADD silencing gave a similar effect as the knockdown of RAP80, a protein important in NHEJ repair. To evaluate whether TRADD is critical for recruitment of repair factors at DNA break sites, we laser-microirradiated in control and TRADD-KD U2OS cells, and stained with 53BP1 and Ku70/80 antibodies for NHEJ or with RPA32 and RAD51 antibodies for HR, respectively in control and TRADD-KD U2OS cells. NHEJ repair factors were dramatically diminished at laser strips after microirradiation, whereas localization of HR pathway factors did not vary at DNA breaks (Fig. 3c–f). As expected, γH2AX recruitment patterns did show alterations in TRADD knockdown cells, and overall fluorescence was increased when quantitated (Fig. 3c and e). However, the Ku70/80 complex, a major factor for NHEJ binding to DNA double-strand break ends, did not accumulate at DNA break sites in TRADD KD cells, and there was also a significant decrease in 53BP1 recruitment at these sites (Fig. 3c and d). The HR factors RPA32 and RAD51 were not decreased at the microirradiation strips in the TRADD-deficient cells (Fig. 3e and f). This is consistent with the fact that GFP-TRADD colocalized with 53BP1 DNA break sites in the mCherry-LacI-FokI endonuclease system. In the absence of DNA damage, the majority of Myc- TRADD was localized in the cytosol rather than in nucleus, and then some Myc-TRADD translocated into the nucleus upon DNA damage and colocalized with the mCherry-FokI signal (Figure S3a). We next examined foci formation of repair factors, 53BP1 (NHEJ) and RPA32 (HR) after treatment with the DNA double-strand break drug phleomycin. The foci formation of 53BP1, but not RPA32, was also reduced at γH2AX foci in TRADD KD cells treated with phleomycin (Figure S3b and c). To exclude the possibility that TRADD depletion affected the expression of repair factors themselves, we monitored the levels of repair factors, 53BP1, Ku70/80, RPA32, and Rad51. None of these factors show any alterations in protein expression upon TRADD depletion (Fig. 3g). These findings suggest that TRADD mainly facilitates NHEJ repair rather than the HR repair of DNA breaks.

### Deletion of TRADD sensitizes cells to DNA damaging agents

**Figure 4:**
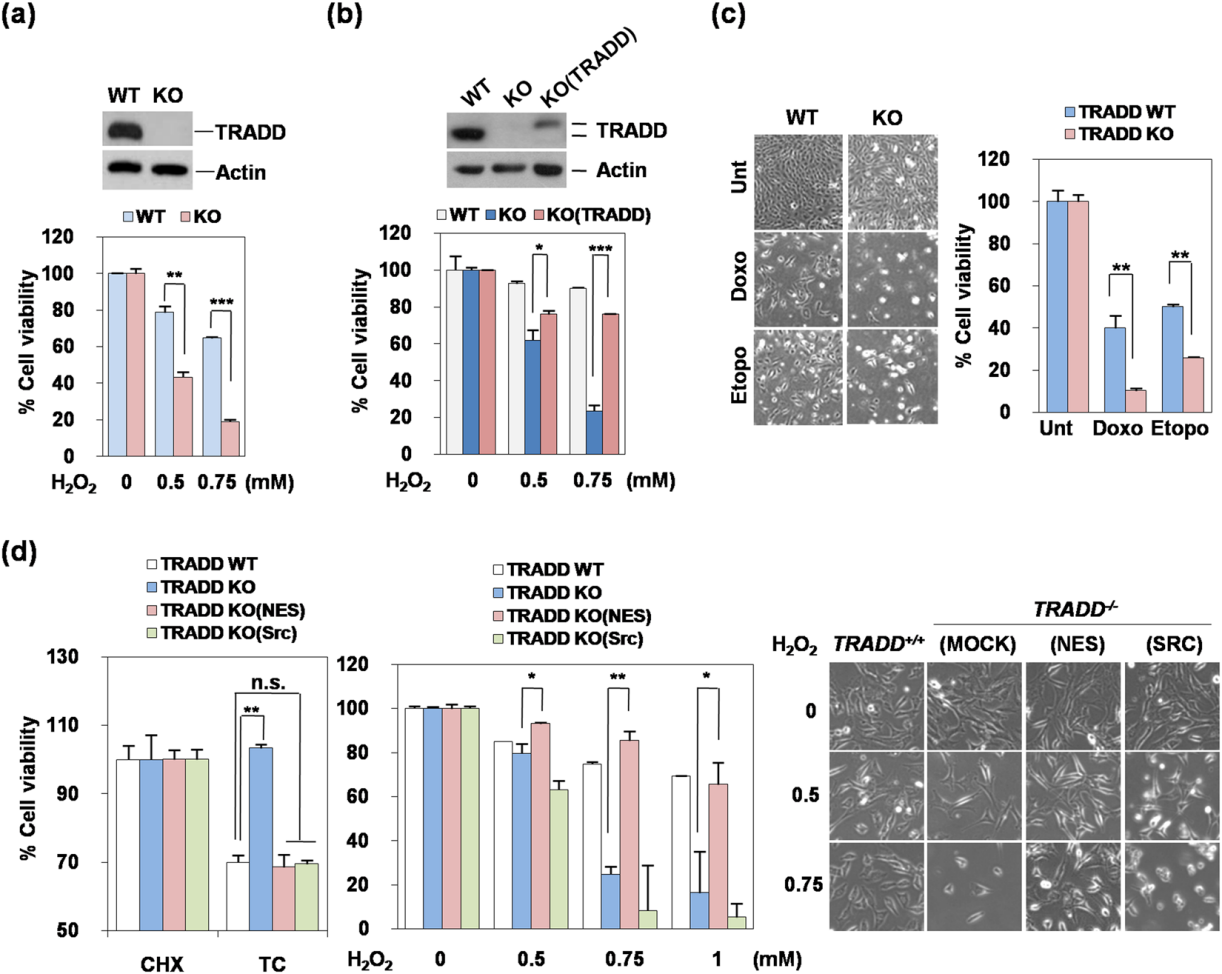
Deletion of TRADD sensitizes to DNA damage agents-induced cell death. (**a**) TRADD^+/+^ (WT) and TRADD^−/−^ (KO) MEFs were treated with H_2_O_2_ for 24 hours and then cell viability was analyzed by MTT assay. **P < 0.01; ***P < 0.001 (Student’s t-test). (**b**) TRADD^+/+^ (WT), TRADD^−/−^ (KO), and TRADD^−/−^ mTRADD (KO-TRADD) cells were treated with H_2_O_2_ for 24 hours. Cell viability was analyzed by MTT assay. *P < 0.05; ***P < 0.001 (Student’s t-test). (**c**) Representative images from treatment with DNA-damaging agents in TRADD wild type and knockout MEFs. TRADD^+/+^ (WT) and TRADD^−/−^ (KO) MEFs were treated with doxorubicin (Doxo) and etoposide (Etopo) for 24 hours and then cell viability was analyzed by MTT assay. **P < 0.01 (Student’s t-test). (**d**) Representative images rescued with TRADD WT, KO, KO (NES), or KO (Src) in knockout MEFs of TRADD. MTT assay of TRADD WT, KO, KO (NES), and KO (Src) MEFs treated with H_2_O_2_ and TNF (30 ng/mL) plus CHX (2.5 μg/mL) for 24 hours. *P < 0.05; **P < 0.01; n.s., not significant (Student’s t-test).

Defects in genes responsible for DNA damage repair frequently cause cell cycle arrest and cell death^18^. We therefore investigated whether depletion of TRADD would sensitize cells to DNA damage agents. Indeed, TRADD^−/−^ MEFs were more sensitive to H_2_O_2_ treatment, but were completely resistant to TNF (Figs 4a and S4a,b). Consistent with these data, TRADD KD HeLa cells were more sensitive to H_2_O_2_ treatment as well (Figure S4c). Reconstitution of TRADD in TRADD^−/−^ MEF reduced H_2_O_2_-induced cell death compared to TRADD^−/−^ MEFs (Fig. 4b). As shown in Fig. 4c, doxorubicin- and etoposide-induced cell death was also increased in TRADD^−/−^ MEFs, indicating that defects in DNA damage repair by depletion of TRADD also causes sensitization of cell death induced by DNA-damaging agents.

To verify the role of nuclear localization of TRADD on cell death, MEFs expressing various TRADD constructs were treated with H_2_O_2_ to induce cell death. As expected, TRADD^−/−^ MEFs were completely resistant to TNF-induced cell death, whereas they were highly sensitive to H_2_O_2_ to induced-cell death (Fig. 4d). Expression of NES mutant-TRADD made cells sensitive to TNF-induced cell death, but protected cells from H_2_O_2_; while the plasma membrane-anchored Src-myr TRADD was able to reconstitute TNF toxicity in TRADD^−/−^ MEFs, but was unable to protect cells from H_2_O_2_ (Figs 4d and S4d). Nuclear TRADD was necessary to inhibit persistent γH2AX accumulation in TRADD knock-out MEF cell lines (Fig. 2e). Our data indicate that nuclear TRADD has an important nuclear role in survival in response to DNA damage. The accumulation of aberrant unrepaired DNA damage such as DSBs in TRADD deficient cells is likely to result in cell death, consistent with what is previously known about the toxicity of DSBs^18^.

### Deficiency of TRADD leads to ROS accumulation and prolonged JNK activation

**Figure 5:**
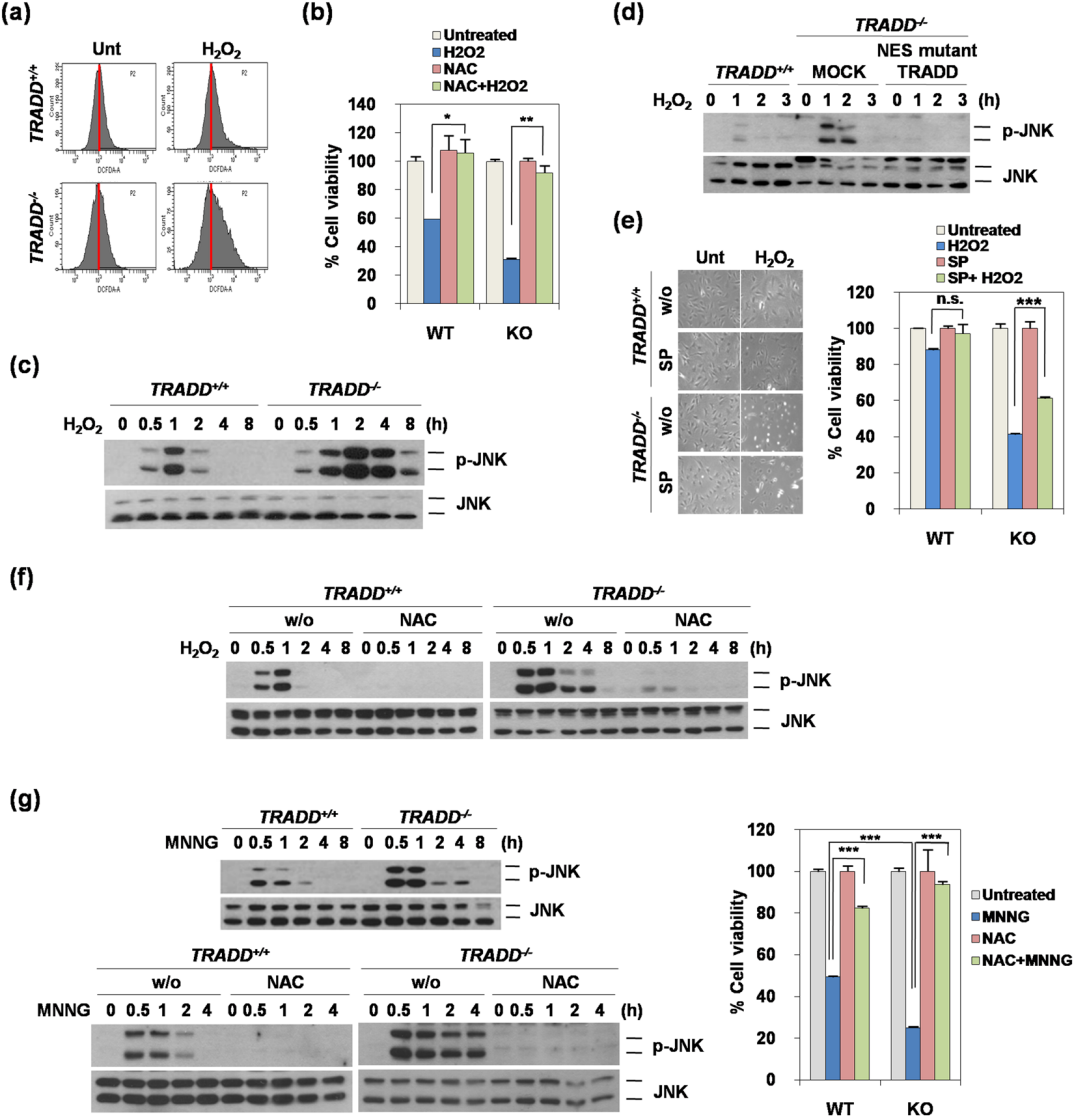
TRADD induces ROS accumulation and prolonged JNK activation. (**a**) TRADD^+/+^ and TRADD^−/−^ MEFs were pretreated with H_2_O_2_ (0.5 mM) for 4 hours followed by treatment with H_2_DCFDA (10 μM) for 30 minutes. ROS levels were measured by flow cytometry. (**b**) MTT assays of TRADD^+/+^ (WT) and TRADD^−/−^ (KO) MEFs treated with NAC (1 mM), H_2_O_2_ (0.5 mM), or NAC (1 mM) plus H_2_O_2_ (0.5 mM) for 24 hours, respectively. *P < 0.05; **P < 0.01 (Student’s t-test). (**c**) Western blotting of lysates from TRADD^+/+^ and TRADD^−/−^ MEFs treated with H_2_O_2_ (0.5 mM) for indicated time periods. (**d**) Western blotting of lysates from TRADD^+/+^, TRADD^−/−^ and TRADD^−/−^ (NES-mutant TRADD) MEFs treated with H_2_O_2_ (0.5 mM) for indicated time periods. (**e**) TRADD^+/+^ and TRADD^−/−^ MEFs were pretreated with JNK inhibitor (SP, 20 μM) for 30 minutes followed by treatment with H_2_O_2_ (0.5 mM) for 24 hours. Cell viability was analyzed by cell morphology and MTT assay. ***P < 0.001; n.s., not significant (Student’s t-test). (**f**) Western blotting of lysates from TRADD^+/+^ and TRADD^−/−^ MEFs pretreated with NAC (1 mM) for 30 minutes followed by treatment with H_2_O_2_ (0.5 mM) for indicated time periods, and then analyzed by western blotting. (**g**) Western blotting of lysates from TRADD^+/+^ and TRADD^−/−^ MEFs treated with MNNG (0.25 mM) for indicated time periods (left upper panel). Western blotting of lysates from TRADD^+/+^ and TRADD^−/−^ MEFs pretreated with NAC (1 mM) for 30 minutes followed by treatment with MNNG (0.25 mM) for indicated time periods, and then analyzed by western blotting (left lower panel). MTT assays of TRADD^+/+^ and TRADD^−/−^ MEFs treated with NAC (1 mM) and MNNG (0.25 mM) for 24 hours (right panel). ***P < 0.001 (Student’s t-test).

DNA damaging agents can increase cellular ROS levels; newly formed ROS can further contribute to DNA damage leading to cell death^19, 20^. To further verify the mechanism of sensitization in impaired DNA repair induced by deficiency of TRADD, we investigated the amount of intracellular ROS. ROS accumulated more readily in TRADD^−/−^ MEFs compared to TRADD^+/+^ MEFs (Fig. 5a) and unsurprisingly, the ROS scavenger, N-Acetyl-L-cysteine (NAC), dramatically decreased cell death in TRADD^−/−^ MEFs, indicating increased ROS in TRADD^−/−^ MEFs contributes to hydrogen peroxide-induced cell death (Fig. 5b). It is well known that ROS induces prolonged JNK activation via inhibition of JNK phosphatase and that prolonged JNK activation contributes to cell death^21^. Thus, we checked whether ROS induces this signal. Indeed, JNK has prolonged activation in TRADD^−/−^ MEFs treated with H_2_O_2_ compared with TRADD^+/+^ MEFs (Fig. 5c). Moreover, the NES-mutant TRADD suppressed JNK activation compared with TRADD^−/−^ MEFs (Fig. 5d). The JNK inhibitor, SP600145, prohibited H_2_O_2_-induced cell death in TRADD^−/−^ MEFs (Fig. 5e), while ROS scavenger treatment inhibited H_2_O_2_-induced prolonged JNK activation (Fig. 5f).

As with other DNA damaging agents, TRADD^−/−^ MEFs are more sensitive to MNNG than TRADD^+/+^ MEFs, with prolonged JNK activation occurring in TRADD^−/−^ MEFs (Fig. 5g, left upper panel). NAC completely blocked MNNG-induced early and prolonged JNK activation occurring in TRADD^−/−^ MEFs, as well as early JNK activation in TRADD^+/+^ MEFs (Fig. 5g, left lower panel). MNNG-induced cell death is also inhibited by NAC (Fig. 5g, right panel). These data suggest that impaired DNA damage induced by deficiency of TRADD affects ROS generation and sustains JNK activation, further potentiating cell death. DNA repair pathways enable cancer cells to survive DNA damage induced by chemotherapeutic treatments, while unrepaired DSBs are highly toxic and can lead to cell death^22^. Taken together, our data suggest that TRADD could be a potential target for DNA damage-based chemotherapeutic agent-induced cancer cell death.

## Discussion

TRADD has a well-established function as an adaptor protein in death receptor signalling in the cytoplasm. However, the function of TRADD in the nucleus is not well understood, though TRADD contains an NLS and an NES and shuttles through the nucleus. Nuclear shuttling of TRADD clearly affects distinct apoptosis mechanisms other than those that are initiated by death receptor apparatus^9^. Chio and colleagues suggested that TRADD shuttles dynamically from the cytoplasm into the nucleus to modulate the interaction between p19Arf and its E3 ubiquitin ligase ULF, thereby promoting p19Arf protein stability and thus tumor suppression^8^.

In this study, we have shown that upon DNA damage, TRADD accumulates in the nucleus and participates in the NHEJ repair process (Figs 3 and S3). Interestingly, an increased basal amount of γH2AX, which is typically an indicator of double strand breaks is detected in TRADD deficient cells in the absence of DNA damaging agents, suggesting that that TRADD may be required for basal repair of DNA damage during basal cellular events, such as cell division. Since TRADD depletion affects the efficiency of NHEJ repair, but does not affect the HR pathway, we speculate that TRADD may serve as mediator to recruit of DNA damage signalling and repair factors to DSBs, especially recruitment of 53BP1 and the Ku70/80 complex, which appear to decrease when TRADD is deficient (Figure S5). Using various TRADD constructs, we have shown that nuclear localization of TRADD is important for its DNA damage response functions. However, the mechanism of DNA damage-induced TRADD nuclear localization, and how it interacts with DNA break sites and NHEJ factors is still unclear.

Post-translational modification (PTM) of TRADD is not well-characterized. Based on a phosphorylation prediction database, we examined the possibility that TRADD could be phosphorylated by various kinases involved in DNA-damage sensing. Interestingly, TRADD is predicted to be phosphorylated by ATM kinase (data not shown). Further study is required to determine if this putative phosphorylation event may play a role in TRADD’s nuclear functions, especially in response to DNA damage and genomic integrity.

Although DNA repair mechanisms seem to be well understood, the cellular response to genotoxic injury induced by conventional chemotherapies and radiotherapy is very complex. In mammals, NHEJ and HR are the two major pathways that repair DNA double-strand breaks (DSBs). The consequences of impairing either NHEJ or HR have been investigated in mice and similar phenotypes are caused by defects in either pathway: hypersensitivity to ionizing radiation and histone deacetylase inhibitors^23–25^. The major proteins involved in NHEJ include the DNA-PKcs and the Ku70/80 heterodimer. These proteins have been reported to be up-regulated in tumors or radiation-resistant cell lines, indicating that NHEJ is likely to have a role in survival and resistance to DNA-damaging chemotherapy^26^. In support of this, Ku70- or 80-depletion sensitizes pancreatic cells to IR, suggesting that it may be a potential target for inhibition in cancer therapy^18^. Thus, the disruption of the DSB repair mechanisms by NHEJ may be of promise in the clinical target for the treatment of various cancers. Given our data, TRADD might be a potential drug target to promote cancer cell death in response to chemotherapeutic DNA-damaging agents.

## Materials and Methods

### Antibodies and chemicals

Actin, TRADD (mouse), Lamin B, RAD51, Myc, and Sp1 antibodies were from Santa Cruz. 53BP1 antibody was from Cell signaling. Hsp90 and RPA32 antibodies were from Abcam. TRADD (human) or γH2AX (phospho-S139) antibodies were from Millipore, Cell signaling and Genetex. Phospho-JNK and JNK antibodies were from Invitrogen. The RIPK1 antibody was from BD transduction. Hydrogen Peroxide (H_2_O_2_), Doxorubicin (Doxo), Etoposide (Etopo), Camptothecin (Cpt), Cisplatin (CDDP), Hydroxyurea (Hu), JNK inhibitor (SP600125), NAC (N-acetyl-I-cysteine), Phleomycin (Phleo), Propidium iodide (PI) and CM-H_2_DCFDA were from Sigma Aldrich.

### Western blot

Cells were lysed in M2 buffer (20 mM Tris at pH 7.0, 0.5% NP-40, 250 mM NaCl, 3 mM EDTA, 3 mM EGTA, 2 mM DTT, 0.5 mM PMSF, 20 mM β-glycerol phosphate, 1 mM sodium vanadate, and 1 μg/mL leupeptin). Equal amounts of cell extracts were resolved by SDS-PAGE and analyzed by immunoblotting.

### Measurement of intracellular ROS

TRADD^+/+^ and TRADD^−/−^ MEFs were treated with H_2_O_2_ for 4 hours. To detect intracellular ROS, we incubated cells with 10 μM CM-H_2_DCFDA for 30 minutes before the end of the indicated treatments. Total ROS was then measured by flow cytometry.

### Cell cycle analysis

Cell cycle was analyzed by PI staining. TRADD knockdown U2OS cell, TRADD^+/+^ and TRADD^−/−^ MEFs were collected and fixed in 75% cold-ethanol. The cells wash in PBS and incubated with RNase (100 μg/mL) for 10 min at 37′C. After RNase incubation, cells were incubated with PI solution (50 μg/mL) for 30 minutes at 37 °C in dark. Cell cycle analysis was analyzed using a flow cytometry.

### Cell culture

TRADD^+/+^ and TRADD^−/−^ MEFs and their culture conditions have been previously described^2^. Murine stable cell lines were established by transfection with GFP-wild type TRADD, GFP-cytoplasmic (Cyto) TRADD, GFP-nuclear export mutant (NES) TRADD, GFP-Src-myristoylated TRADD (Src)^9^ and then selected with G418/Neomycin. Mutant plasmid expression in TRADD^−/−^MEFs and HeLa was confirmed by western blotting and immunofluorescense. MEF, HeLa, U2OS, and U2OS 2-6-3 cells were maintained in DMEM medium supplemented with 10% fetal bovine serum (FBS) and penicillin-streptomycin.

### Cell viability assay

Cell viability was determined using a tetrazolium dye colorimetric test (MTT), with absorbance read at 570 nm, or were performed using a Cell Titer-Glo Luminescent Cell Viability Assay kit (Promega, G7570) according to manufacturer’s instructions.

### NE-PER nuclear and cytoplasmic extraction

For nuclear and cytoplasmic fractionation, cells were washed twice in ice-cold PBS. Nuclear and cytoplasmic extraction was performed using a NE-PER nuclear and cytoplasmic extraction kit (Thermo, #7833) according to manufacturer’s instruction. Equal amounts of protein were loaded in SDS-PAGE. Histone H3 and Sp1 was used for nuclear fraction normalization, while Hsp90 was used for cytoplasmic fraction normalization.

### Non-homologous end-joining (NHEJ) and homologous recombination (HR) repair assay

U2OS GFP reporter cell lines, U2OS-EJ5-GFP (NHEJ) and U2OS-DR-GFP (HR), were transfected with the indicated siRNAs by Lipofactamine RNAiMAX (Invitrogen). On the following day, I-*Sce*I endonuclease was delivered to the cells, and 72 hrs later, they were assayed for GFP-positive cells by flow cytometry (BD Biosciences)^27^.

### Laser microirradiation

For the recruitment of NHEJ or HR repair factors at DNA break sites, U2OS cells were processed as described previously^15^.

### FokI assay

For the recruitment of GFP-TRADD at single DNA double-strand break site (sDSB), mCherry-LacI-FokI and GFP-TRADD wild type, GFP-TRADD Src mutant, or GFP empty vector were cotransfected into U2OS 2-6-3 cell lines. After 48 hrs, fixed cells were stained with DAPI. Furthermore, to detect the localization of Myc-TRADD at sDSB, transfected cells were stained with anti-53BP1 and anti-Myc antibodies. Colocalization of mCherry-FokI and GFP-TRADD at sDSB was analyzed with a Nikon confocal microscope A1^15^.

### Small interfering RNA knockdown

HeLa and U2OS cells plated in 60 mm dish. The cells were transfected with 100 pmol of TRADD (Bioneer) or a non-targeting control RNAi oligo (Bioneer) using Lipofectamine 2000 (Invitrogen, Inc.). After 24 hrs, cells were seeded in 6 well or 12 well plates and then treated with a DNA-damage agent. The knockdown efficiency of TRADD confirmed by western blotting. The target sequences in TRADD mRNA are 5′-CGAAUGUUAAGCAAUGAUAUU-3′ for siRNA#1 and 5′-CAAUGAUAAUAAAGUAUAAUU-3′ for siRNA#2; The target sequences in RAP80 mRNA are 5′-GAAGGAUGUGGAAACUACC-3′; The target sequences in BRCA1 mRNA are 5′-UCACAGUGUCCUUUAUGUA-3′.

### Confocal microscopy

Cells were seeded in a coverglass slide chamber and treated with designated agents, coverslide fixed for 10 min in 4% paraformaldehyde, and then stained with γH2AX (phospho-S139), 53BP1 and RPA32. Cells were mounted with 10 mg/ml DAPI in aqueous mountant (Dako, Denmark) and viewed by confocal microscopy.

### Statistics

Statistical analysis was performed using ANOVA and a Student’s t-test. For all analyses *P* < 0.05 was considered statistically significant, and *P < 0.05, **P < 0.01, ***P < 0.001.

## Electronic supplementary material

Supplementary information
