# Supplementary material for: Nuclear TRADD prevents DNA damage-mediated death by facilitating non-homologous end-joining repair
Source: Sci Rep. 2017 Jun 13;7:3332. doi: 10.1038/s41598-017-03211-z (PMC5469829; doi:10.1038/s41598-017-03211-z)

Supplementary information

Nuclear TRADD prevents DNA damage-mediated death by facilitating non-homologous end-joining repair

Gi-Bang Koo[1, 2, #], Jae-Hoon Ji[3, #], Hyeseong Cho[1, 2, 3], Michael J. Morgan[4] and You-Sun Kim[1, 2, *]

[1] Department of Biochemistry, Ajou University School of Medicine, Suwon, Gyeonggi, 16499, Republic of Korea
[2] Department of Biomedical Sciences, graduate School, Ajou University, Suwon, Gyeonggi, 16499, Republic of Korea
[3] Genomic Instability Research Center, Ajou University School of Medicine, Suwon, Gyeonggi, 16499, Republic of Korea
[4] Department of Pharmacology, University of Colorado School of Medicine, Aurora, Colorado, 80045, USA

[#] These authors contributed equally to this work.

* To whom correspondence should be addressed. Tel:82-31-219-4509; Fax: 82-31-219-4530;Email:yousunkim@ajou.ac.kr

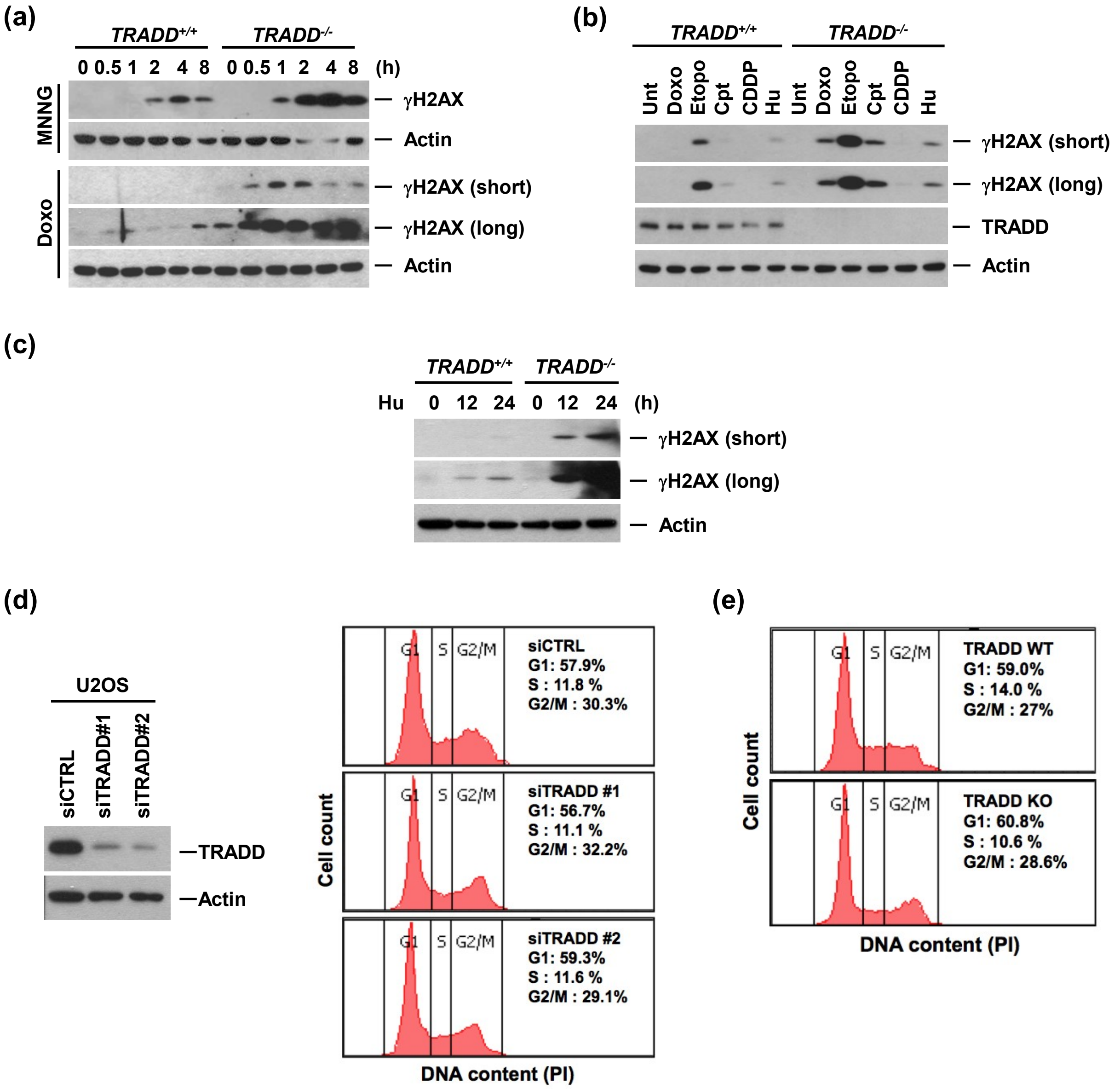

Supplementary figure S1. TRADD regulates DNA repair following treatment of DNA damage agents. (**A**) Western blotting analysis shows γH2AX and Actin in TRADD$^{+/+}$ and TRADD$^{-/-}$ MEF cells treated with MNNG (0.25 mM) or doxorubicin (2.5 μM), respectively. (**B**) Impaired DDR (DNA damage response) in TRADD$^{-/-}$ MEFs is a common phenotype. Deficiency of TRADD induces unrepaired DNA damage compared with wild type cells in response to various DNA damaging agents (Doxo 2.5 μM, Etoposide 25 μM, Cpt 2.5 μg/mL, CDDP 25 μM, Hu 2.5 mM) for 2hrs. (**C**) TRADD$^{+/+}$ and TRADD$^{-/-}$ MEF cells treated with Hu 2.5mM for indicated times. (**D**) Cell cycle analysis of TRADD knockdown into U2OS cells. The cells were transfected with siCTRL, siTRADD #1, and siTRADD #2 for 48 hrs. Cell cycle analysis was performed by PI staining. Western blotting shows TRADD knockdown efficiency in U2OS cells. (**E**) Cell cycle analysis by PI staining of TRADD$^{+/+}$ and TRADD$^{-/-}$ MEF cells.

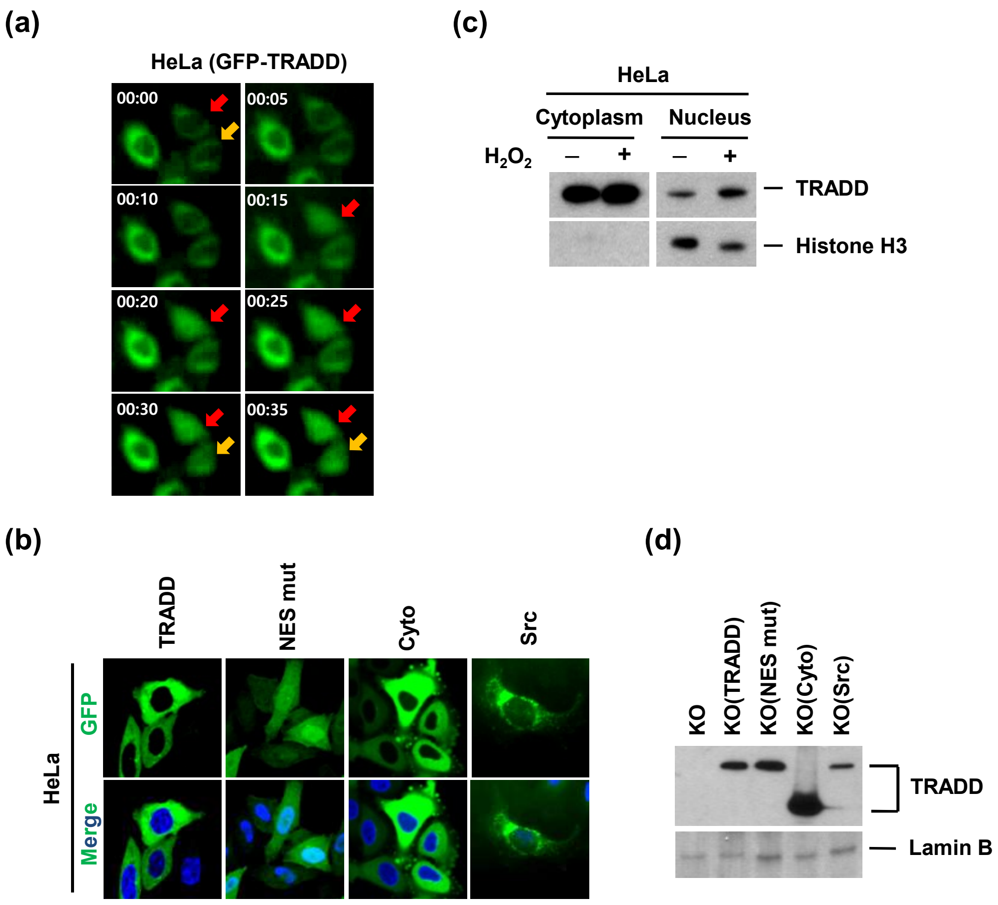

Supplementary figure S2. Cytoplasmic TRADD is translocated into nucleus upon DNA damage. (**A**) HeLa cells were transfected with GFP-TRADD and treated with H_2O_2 (0.5 mM) for indicated times (minutes). Live cell images were analyzed by JuLI stage (live cell imaging system). (**B**) Localization of TRADD constructs into HeLa cells. Cells were transfected with wild type-TRADD (TRADD), (nuclear export sequence) mutant-TRADD (NES mut), cytoplasmic-TRADD (Cyto), and Src-myristoylated-TRADD (Src). (**C**) HeLa cells were treated with H_2O_2 (0.5 mM, 2 hrs). Cells were fractionated into cytoplasmic and nuclear fractions using an NE-PER fractionation kit, and then analyzed by western blotting. (**D**) Western blotting shows that expression of TRADD constructs in TRADD$^{-/-}$ MEFs.

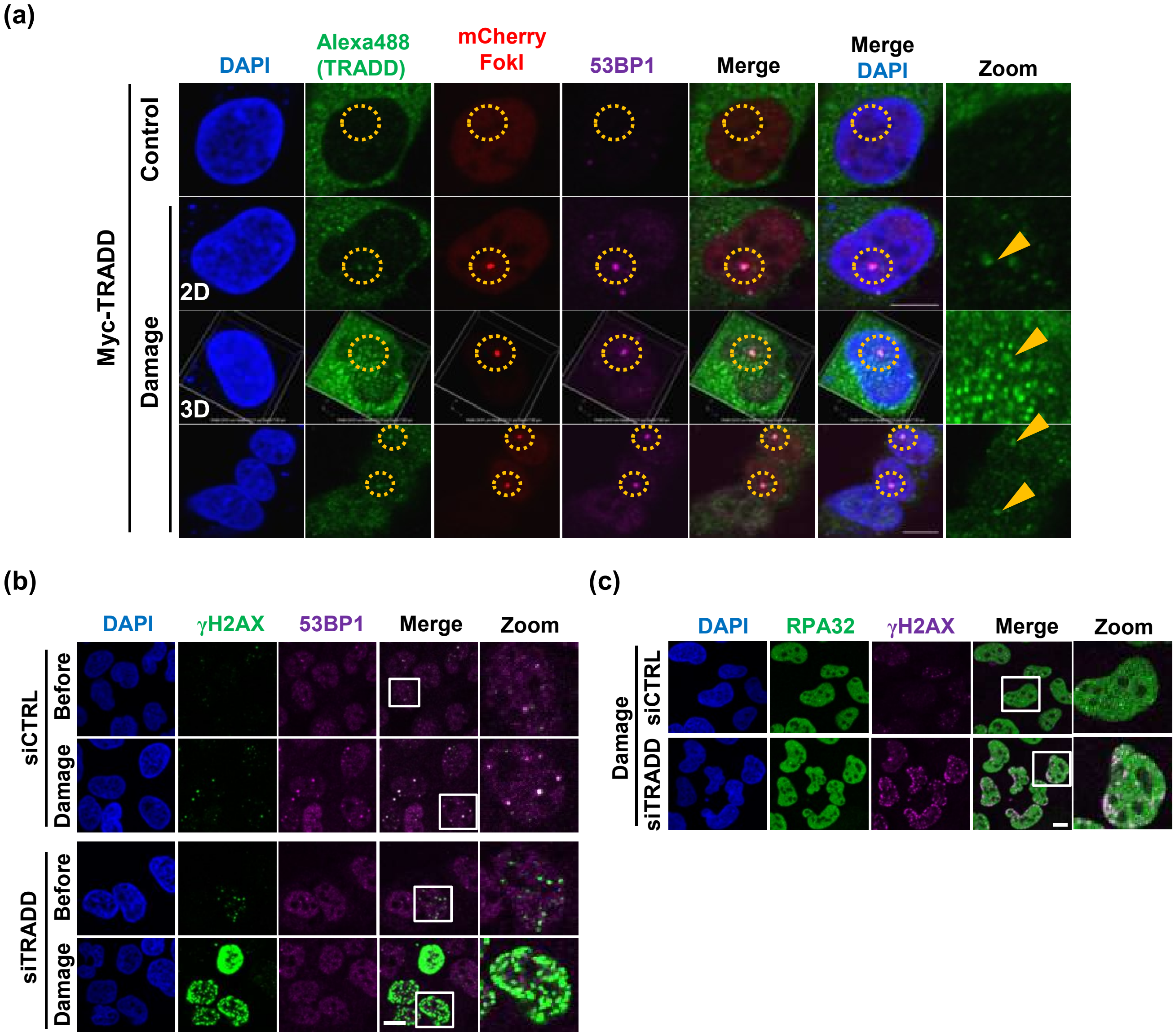

Supplementary figure S3. TRADD is translocated at DSB site and downregulates 53BP1 foci formation, but not RPA32 foci formation upon DNA damage.

(**A**) Colocalization of Myc-TRADD and mCherry-FokI at single DNA double-strand break site. Constructs of Myc-TRADD and mCherry-FokI were cotransfected into U2OS 2-6-3 cell lines. After 48 hrs, cells were fixed and stained with anti-53BP1 and anti-Myc antibodies. DAPI was used for nuclear staining. Images were analyzed confocal microscope (Nikon A1). 2D, two-dimensional analysis for images; 3D, three-dimensional analysis for images. Scale bar, 10 μm. (**B**,**C**) After 48 hrs transfection with TRADD targeting siRNAs into U2OS cell lines, cells treated with phleomycin (50 μg/mL, 1 hr). After wash out with fresh medium, endogenous 53BP1 (B) and RPA32 (C) were stained with each antibody at DNA break sites, 3 hrs later. γH2AX was used as a DNA damage marker at DNA break sites.

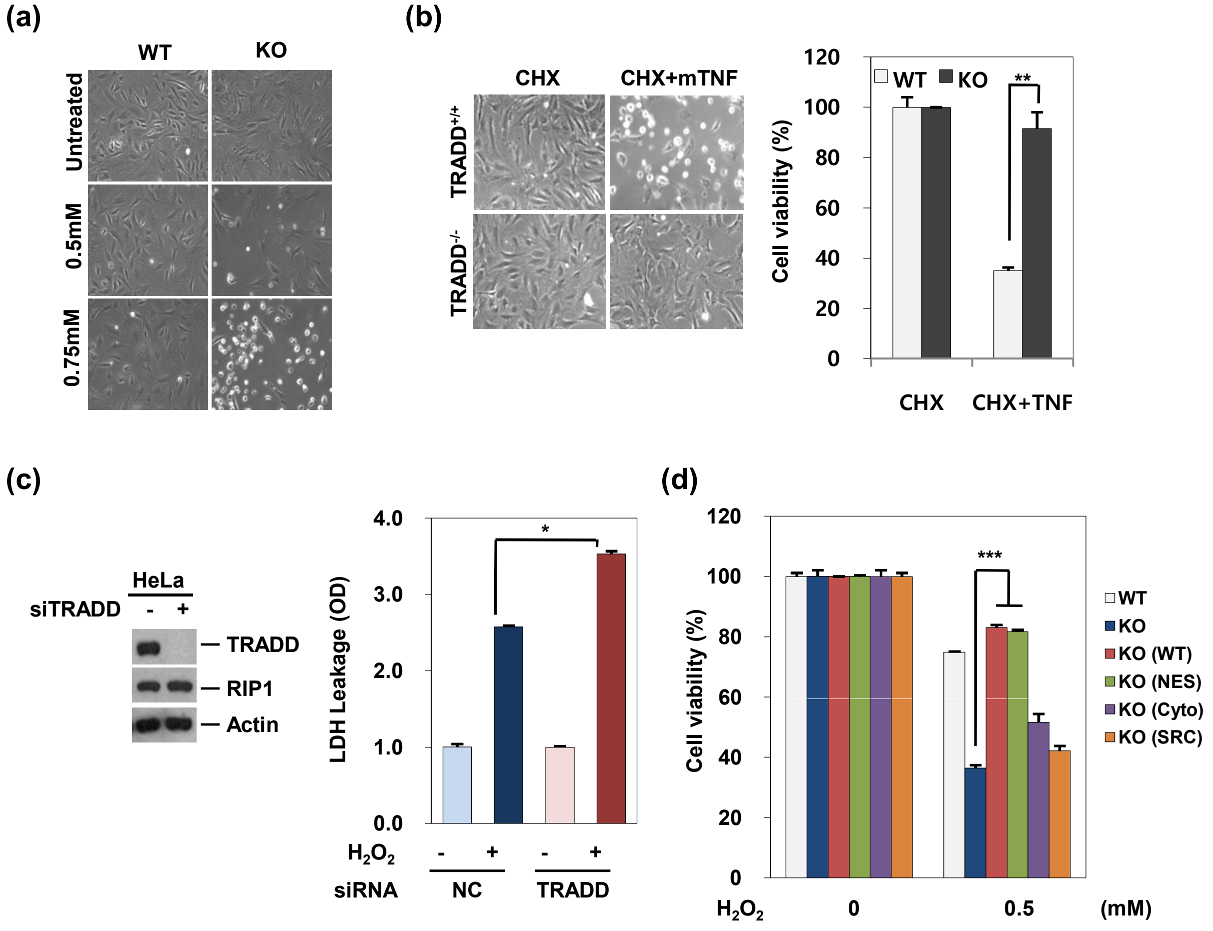

Supplementary figure S4. Deficiency of TRADD sensitizes DNA damage-induced cell death.
(**A**) Phase contrast images of TRADD$^{+/+}$ (WT) and TRADD$^{-/-}$ (KO) MEFs treated with H_2O_2 (24 hrs). (**B**) Phase contrast images (left panel) and MTT viability assay (right panel, Error bars: +/− S.E.M.) of TRADD$^{+/+}$ and TRADD$^{-/-}$ MEFs treated with CHX (2.5 µg/mL) plus TNF (30 ng/mL, 24 hrs). **P < 0.01 (Student`s t-test). (**C**) HeLa cells were transfected with siRNA TRADD or siRNA control (NC), respectively. After 48 hrs, the cells were treated with $H_2O_2$ for 24 hrs. Cell viability was analyzed by LDH assay. *P < 0.05 (Student` s t-test). (**D**) MTT assay of TRADD$^{+/+}$ (WT), TRADD$^{-/-}$ (KO), TRADD$^{-/-}$ (KO (WT)), TRADD$^{-/-}$ (KO (NES mutant TRADD : NES)), TRADD$^{-/-}$ (KO (Cytoplasmic mutant TRADD : Cyto)) and TRADD$^{-/-}$ (KO (Src-TRADD:SRC)) MEFs treated with H_2O_2 for 24 hrs. ***P < 0.001 (Student`s t-test).

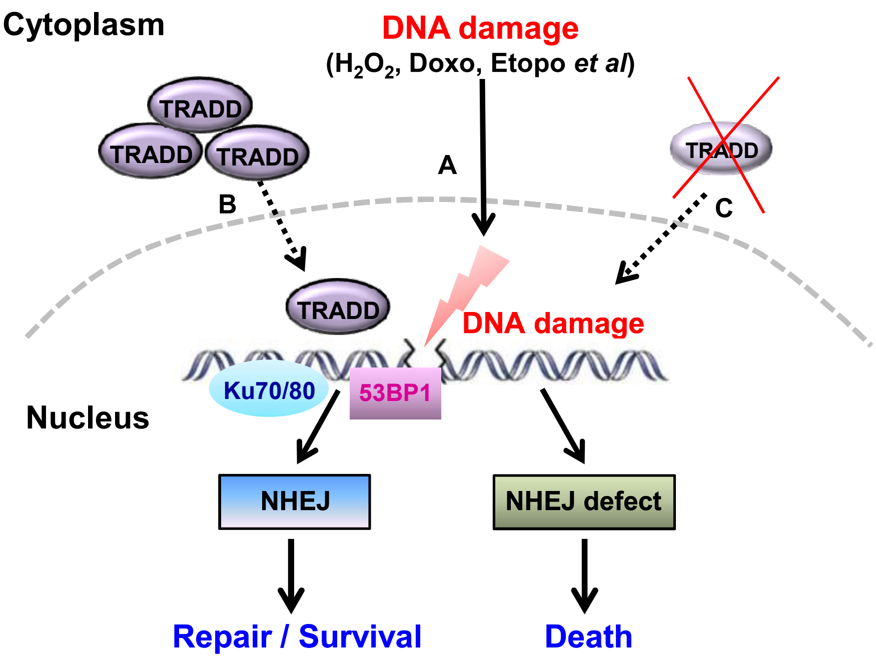

Supplementary figure S5. Proposed model.
(**A**,**B**) DNA damage promotes translocation of TRADD from cytoplasm to the nucleus. Nuclear TRADD facilitates DNA repair through promoting of non-homologous end-joining (NHEJ) repair. (**C**) If broken DNA is impaired by deficiency of TRADD, cells are more sensitive to DNA damage-induced cell death.

Figure raw data.

Figure 1

(a)

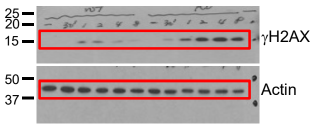

(d)

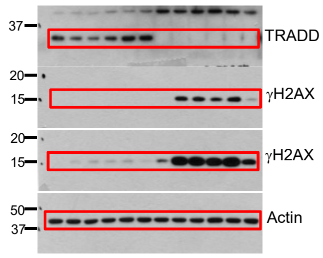

(e)

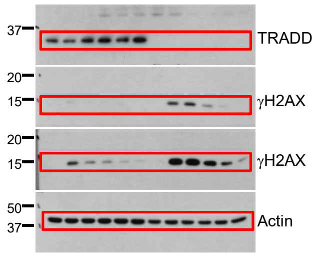

(g)

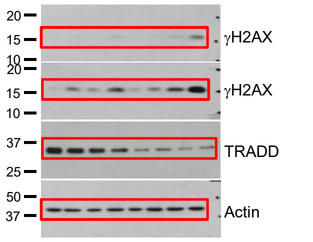

(h)

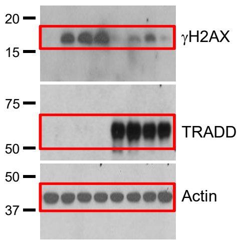

Figure 2

(d)

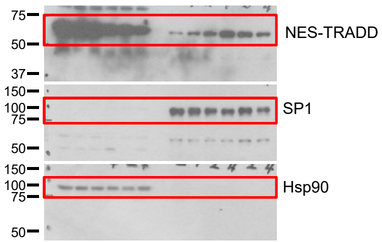

(e)

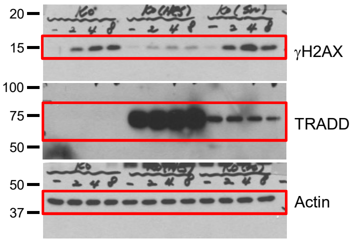

Figure 3

(a)

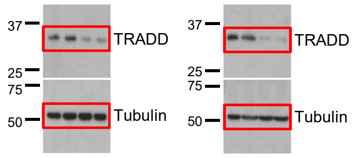

(g)

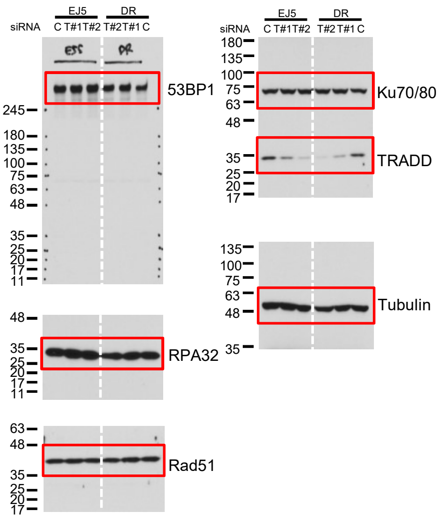

Figure 4

(b)

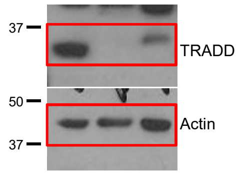

(d)

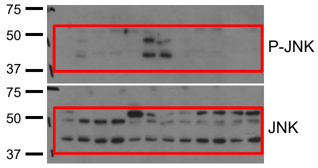

Figure 5

(C)

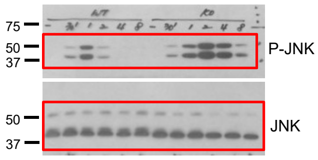

(g)

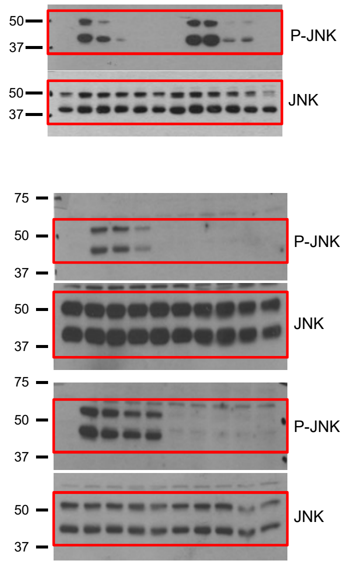

(f)

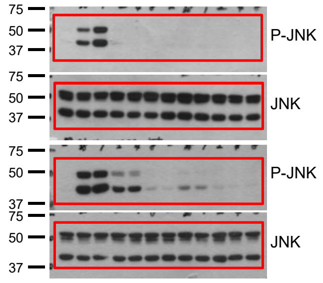

Supplement: Supplementary file 1 — Supplementary information [file 41598_2017_3211_MOESM1_ESM.pdf]
